# Regulation of mRNA Translation Is a Novel Mechanism for Phthalate Toxicity

**DOI:** 10.1371/journal.pone.0167914

**Published:** 2016-12-19

**Authors:** Jun Ling, Zenaida P. Lopez-Dee, Colby Cottell, Laura Wolfe, Derek Nye

**Affiliations:** 1 Department of Basic Sciences, The Commonwealth Medical College, Scranton, Pennsylvania, United States of America; 2 Department of Sciences, Marywood University, Scranton, Pennsylvania, United States of America; 3 Department of Biology, Wilkes University, Wilkes-Barre, Pennsylvania, United States of America; Qingdao Agricultural University, CHINA

## Abstract

Phthalates are a group of plasticizers that are widely used in many consumer products and medical devices, thus generating a huge burden to human health. Phthalates have been known to cause a number of developmental and reproductive disorders functioning as endocrine modulators. They are also involved in carcinogenesis with mechanisms less understood. To further understand the molecular mechanisms of phthalate toxicity, in this study we reported a new effect of phthalates on mRNA translation/protein synthesis, a key regulatory step of gene expression. Butyl benzyl phthalate (BBP) was found to directly inhibit mRNA translation *in vitro* but showed a complicated pattern of affecting mRNA translation in cells. In human kidney embryonic cell (HEK-293T), BBP increased cap-dependent mRNA translation at lower concentrations but showed inhibitory effect at higher concentrations. Cap-independent translation was not affected. On the other hand, mono (2-ethylhexyl) phthalate (MEHP) as a major metabolite of another important phthalate di (2-ethylhexyl) phthalate (DEHP) inhibited both can-dependent and -independent mRNA translation *in vivo*. In contrast, BBP and MEHP exhibited an overall promoting effect on mRNA translation in cancer cells. Mechanistic studies identified that the level and phosphorylation of eIF4E-BP (eIF4E binding protein) and the amount of eIF4GI in eIF4F complex were altered in accordance with the effect of BBP on translation. BBP was also identified to directly bind to eIF4E, providing a further mechanism underlying the regulation of mRNA by phthalate. At the cellular level BBP inhibited normal cell growth but slightly promoted cancer cells (HT29) growth. Overall, this study provides the first evidence that phthalates can directly regulate mRNA translation as a novel mechanism to mediate their biological toxicities.

## Introduction

Phthalates are a group of diesters of 1, 2-benzenedicarboxylic acid that widely used as plasticizers and solvents in a broad range of consumer products, construction materials, food package, child products, cosmetic products and medical devices [1, 2]. The increasing contamination of phthalate affects a large population of humans and causes a huge health burden [3]. Ingested phthalates are hydrolyzed to their corresponding monoester in intestine and parenchyma, therefore becoming the active forms of phthalate *in vivo* [4]. In humans, toxicity of phthalates has been inferred in at least 20 diseases, including endocrine and reproductive disorders, liver, cardiovascular, and urologic diseases [5]. As endocrine disruptors, phthalates cause “phthalate syndrome” that affects male reproductive tract abnormalities (e.g., shortened anogenital distance, hypospadias, and cryptorchidism) [6, 7]. The reduction of fetal testosterone and insulin-like growth factor-3 (Insl-3) by phthalates is an underlying molecular mechanism [8]. In female, phthalates also cause prolonged oestrous cycle, delayed ovulation, and smaller preovulatory follicles [9, 10]. The other major toxicity of phthalate is liver carcinogenicity [11], which is mainly through the interaction with the peroxisome proliferators-activated receptor (PPAR)α [12]. Other PPARα-independent pathways (e.g., signal transduction) are also involved in mediating the phthalate toxicity to liver [13]. Phthalates are also demonstrated to correlate with other cancers, such as breast and prostate cancer [14, 15]. Molecular mechanism studies have been more focused on the regulation of gene transcription [16, 17] and the functioning through nuclear receptors, such as PPARs, estrogen receptor [18], androgen receptor [19], and glucocorticoid receptor [20]. mRNA translation/protein synthesis as a critical regulatory step of gene expression has been much less studied. However, mRNA translation machinery is localized in cytoplasm and more sensitive to environmental stimuli than gene transcription [21], thus rationalizing this study to be significant by directly examining the effects of phthalates on mRNA translation.

mRNA translation is a fundamental and regulatory step of gene expression that determines the cellular proteome [21]. Initiation is a rate-limiting step of translation, which is also the node of regulation [22]. There are about 16 eIFs (over 30 proteins including subunits) as the key regulators of translation initiation [23], among which phosphorylation [24], proteolytic modification [25], protein-protein interaction [26], protein-RNA interaction [27], and isoforms [28] are all involved in regulating eIF activities. In addition, mRNA translation is also regulated by cap-dependent and -independent modes [29], and miRNA [30]. All of these types of regulation control the global and selective mRNA translation, therefore systematically determining the physiological functions of cells.

There were limited early studies on the effect of phthalates on protein synthesis. Most of those studies were performed in animals. MEHP was found to inhibit protein synthesis in rat hepatocytes by [^3^H]-leucine incorporation experiment [31]. However, in the primary culture of Sertoli cells, MEHP was not effective on translation [32]. On the other hand, a rat liver experiment demonstrated that DEHP promoted protein synthesis [33], which was somewhat correlated with the dual effects we observed in HEK293 cells. There were also a few reports about the regulation of individual protein synthesis by phthalates. Hepatic carnitine palmitoyltransferase (CPT) synthesis was increased by DEHP treatment in rats [34, 35]. One recent study identified that ribosomal protein synthesis is impaired by BBP, therefore indirectly affecting the overall mRNA translation [36]. Another study revealed that monobutyl phthalate can target miRNA-200c to regulate mRNA translation [37]. Those studies provided evidence that phthalates might regulate mRNA translation. However, there is no study to address if phthalates can directly target mRNA translation and which translational steps are affected. Nevertheless, all of these high quality researches support our idea in this study to directly investigate this topic.

In this study, two commonly used phthalates were used to investigate the effects of phthalates on mRNA translation. BBP has been used worldwide as a plasticizer in polyvinyl chloride (PVC) products, generating a serious environmental contamination and causing a range of human health problems with teratogenic and possible carcinogenic activities [38]. DEHP as one of the most commonly produced and used phthalates in the US also causes a large array of human health problems [39]. By using various translational assays, we identified that both phthalates inhibited mRNA translation *in vitro* but showed different patterns on the regulation of mRNA translation in normal and cancer cells. Differential effects on cap-dependent and -independent translation and the overall effect on cell growth were also studied.

## Materials and Methods

### Materials

Butyl benzyl phthalate (BBP, Cat#308501) and DL-mono-1-methylhexyl phthalate (MEHP, S879479) were purchased from Sigma-Aldrich. All other chemicals used in this research are at analytical grade. Human kidney embryonic cell (HEK-293T) and colon cancer cell (HT-29) were purchased from ATCC. DMEM media and fetal bovine serum (FBS) were bought from Atlanta Biologicals, and McCoy’s 5A media was bought from ATCC. Transfection reagent (TransIT-2020) was from Mirus. *in vitro* translation system—rabbit reticulocyte lysate (Cat#L4960) and luciferase assay kits (Cat# E2610 for firefly luciferase; Cat#E1910 for dual luciferase assay) were bought from Promega. 7-methyl-GTP Sepharose 4B affinity resin was bought from GE Healthcare. Biosensor chip CM5 and amine coupling kit were purchased from GE Healthcare. WST-1 cell proliferation assay kit (Cat#MK400) was purchased from Takara/Clontech. The bicistronic reporter pRMF with c-myc IRES to measure cap-dependent and -independent translation was a gift from Dr. Anne E. Willis (University of Nottingham, UK). Antibodies to eIF4GI raised in rabbit against the peptide 920–1396 and to eIF4E against peptide 199–217 were provided by Dr. Simon J. Morley (University of Sussex, UK). Other antibodies against eIFs and actin were from Cell Signaling Technology and Santa Cruz Biotechnology. Infrared labeled secondary antibodies were from LI-COR Biosciences and Rockland Immunochemicals.

### Cell culture, transfection, and treatment

HEK-293T cell was cultured in DMEM media with 10% FBS and HT-29 cell in McCoy’s 5A media with 10% FBS at 37C with 5% CO_2_. Cells at 80% confluency were transfected with luciferase reporter construct using TransIT-2020 transfection reagent according to the manufacture’s protocol for *in vivo* translational activity measurement. BBP or MEHP diluted or dissolved in methanol [40] was directly added to the media to treat the cells. 100-fold concentrated phthalates were used to minimize the perturbation to media conditions.

### Measurement of mRNA translational activity

Rabbit reticulocyte lysate (Promega) was used to measure mRNA translational activity *in vitro*. Low amount of capped and polyadenylated luciferase reporter mRNA (0.5μg/20μl reaction) was used to mimic the regulation of translation *in vivo* [41]. The translation reaction (20μl), containing 50% lysate, 100 mM amino-acid mixture, 1U of RNasin (Promega, 40 U/ml) and the phthalate at different concentrations, was incubated at 30C for 1 hr. The luciferase activity was then measured using the luciferase assay kit on luminometer (GloMax 20/20, Promega).

To measure the *in vivo* translation activity, cells transfected with dual luciferase reporter (pRMF) were treated with phthalates and then lysed by passive lysis buffer (Promega) for the quantitation of Renilla luciferase and firefly luciferase activities using dual luciferase assay kit (Promega). Thus, the cap-dependent and -independent translational activities can be determined simultaneously.

### Western blotting

Cells after treatment were extracted with buffer A (25 mM Tris-HCl pH 7.6, 150 mM NaCl, 0.5% NP-40, 0.5% Sodium deoxycholate, 0.05% SDS and 5 mM β-mercaptoethanol, supplemented with fresh protease inhibitor and phosphatase inhibitor cocktails (Set III, EMD)) to prepare the total cell lysate. Protein concentration was measured using the Bradford assay (Coomassie Plus, Pierce/Thermo Scientific). Equal amounts of protein were separated by SDS-PAGE and transferred onto the nitrocellulose membrane by semi-dry transfer procedure (Bio-Rad). The hybridization conditions and the dilution ratios of primary and secondary antibodies were based on the antibody specifications and the protocol from LI-COR with optimization. The final image was acquired using the Odyssey infrared scanner (LI-COR). Data were analyzed using the Odyssey software 3.0.

### Cell viability assay

Cells were cultured in 96-well plates with 100μl of media per well. After the treatment, 10ul of WST-1 reagent (Clontech) was added and continuously incubated for 2 hrs, and then the formazan formation was quantitated by the absorbance at λ_450nm_ with background subtraction at λ_690nm_. The adjusted absorbance units were used to quantitate cell viability.

### Surface plasmon resonance (SPR) assay

The real-time and label-free SPR technique was used to measure the binding of phthalate to eIF4E. CM5 chip was used to immobilize eIF4E as the ligand according to amine coupling protocol (GE Healthcare). Purified eIF4E was immobilized to Fc2 channel as the assay channel, and the Fc1 channel was equally treated but without protein as the control channel. Phthalate diluted in PBS running buffer was injected through Fc1 and Fc2 channels to measure the binding to eIF4E. The experiment was performed on Biacore X-100 Plus SPR biosensor (GE Healthcare) according to the Biacore’s protocols and our previous study [42].

## Results

### BBP directly inhibits mRNA translation *in vitro*

**Fig 1:**
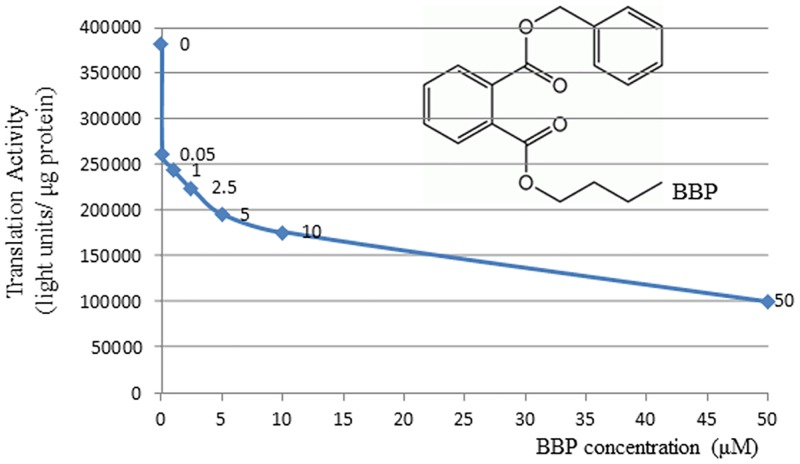
BBP inhibits mRNA translation *in vitro*. Rabbit reticulocyte lysate system was used to measure the translation activity *in vitro* using luciferase mRNA as the reporter. BBP was added to the final concentrations as indicated. Equal volume of methanol only was used as the control. Treatment with each concentration was repeated three times and the average values were shown. The structure of BBP was inserted in the chart.

To test if BBP has direct effect on mRNA translation, the rabbit reticulocyte lysate (RRL) *in vitro* translation system was used. It was found that mRNA translation was sensitively inhibited by BBP at the concentration as low as 0.05μM. Up to 10μM concentration of BBP, translation was inhibited by 54% (Fig 1). The effect of BBP at each concentration point was normalized by the subtraction of methanol control, thus the nonspecific effect of methanol vehicle can be eliminated. The same is true for the data analysis in the rest of experiments. Since RRL system only contains mRNA translation machinery components, this result clearly indicated that BBP had direct inhibitory effect on mRNA translation rather than affecting gene transcription and other biological pathways.

### Phthalates affect mRNA translation differentially *in vivo*

**Fig 2:**
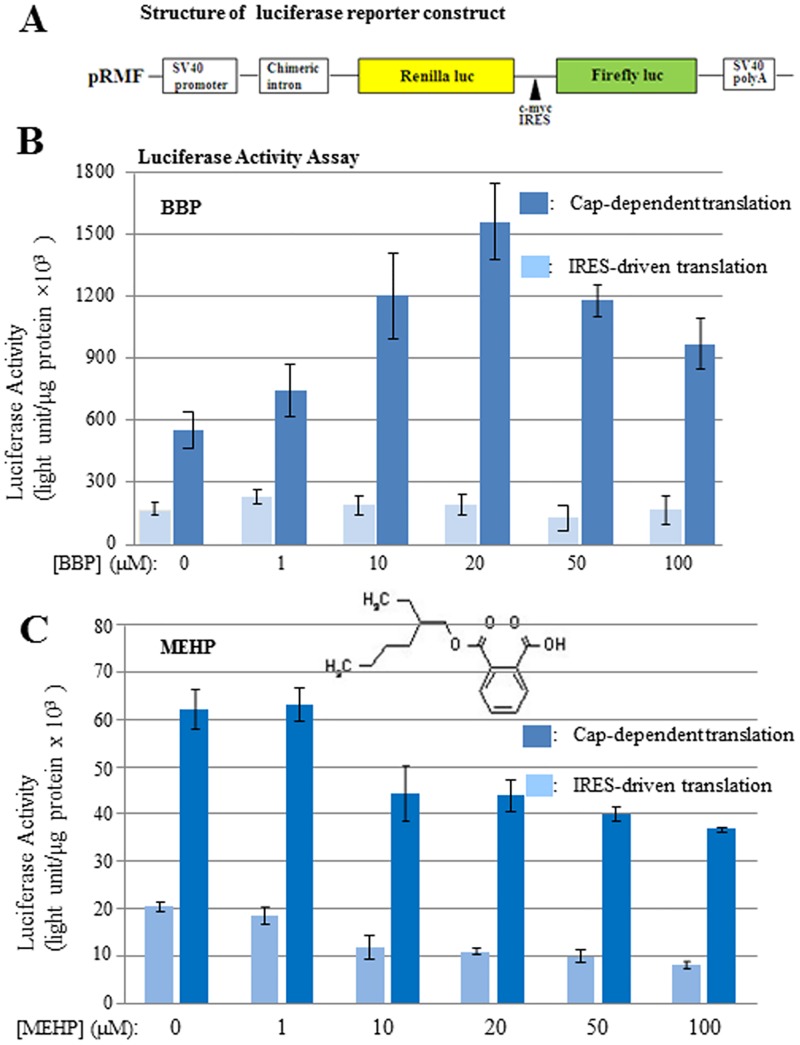
The effects of BBP and MEHP on mRNA translation in HEK-293T cells. HEK-293T cells were grown to 80% confluence and transfected with luciferase reporter. Eight hours later, cells were treated with BBP or MEHP for 24 hrs at the concentrations indicated. Equal volume of vehicle (methanol) was used as the control. Cells were harvested by washing once with cold PBS, and the cell lysate was prepared with PBL buffer (Promega) for the luciferase activity measurement using dual luciferase assay kit (Promega). **(A)** Schematic diagram of the reporter construct used in this experiment and throughout the whole study. **(B)** The effect of BBP on cap-dependent and IRES-driven translation; luciferase activity is the readout of translational activity. **(C)** The effect of MEHP on cap-dependent and IRES-driven translation. The structure of MEHP was inserted in the chart. The experiments were repeated three times, and the means were shown with standard deviations.

To further understand how BBP affects mRNA translation *in vivo*, human HEK-293T cells were tested. Since all phthalate metabolites are eventually secreted through kidney, the results here may shed light on the phthalate toxicity to kidney. To comprehensively evaluate the effect of phthalate on mRNA translation, a reporter gene containing both cap-dependent and cap-independent (IRES-driven) translational elements was used (Fig 2A). By reading *Renilla* luciferase as the activity of cap-dependent translation and firefly luciferase as the activity of IRES-driven translation, it was found that lower concentrations of BBP increased cap-dependent translation, but BBP concentrations higher than 20μM changed to inhibit mRNA translation (Fig 2B). This two-phasic response to BBP indicated complicated mechanisms for BBP to affect mRNA translation in cells. Direct and indirect pathways of phthalate functioning might be all possibly involved. On the other hand, BBP was found to have no effect on IRES-driven mRNA translation (Fig 2B), another mode of mRNA translation representing a small group of mRNAs in human cells. These differential effects suggest that BBP selectively affects a large portion of human genes that are translated through cap-dependent mode, whereas smaller numbers of genes translated through IRES-driven mode are less affected by BBP.

To test if other phthalates express similar effect on mRNA translation *in vivo*, MEHP as an effective metabolite of DEHP was examined. By using the similar experimental procedure as above, it was found that MEHP showed a simple pattern to inhibit both cap-dependent and IRES-driven translation, wherein IRES-driven translation was more sensitive to the inhibition (Fig 2C). This result suggests that different phthalates exhibit different activities to inhibit or promote mRNA translation. Their selectivity on cap-dependent and IRES-driven translation is also different, supporting the hypothesis that different phthalates express various toxicities through different physiological pathways.

**Fig 3:**
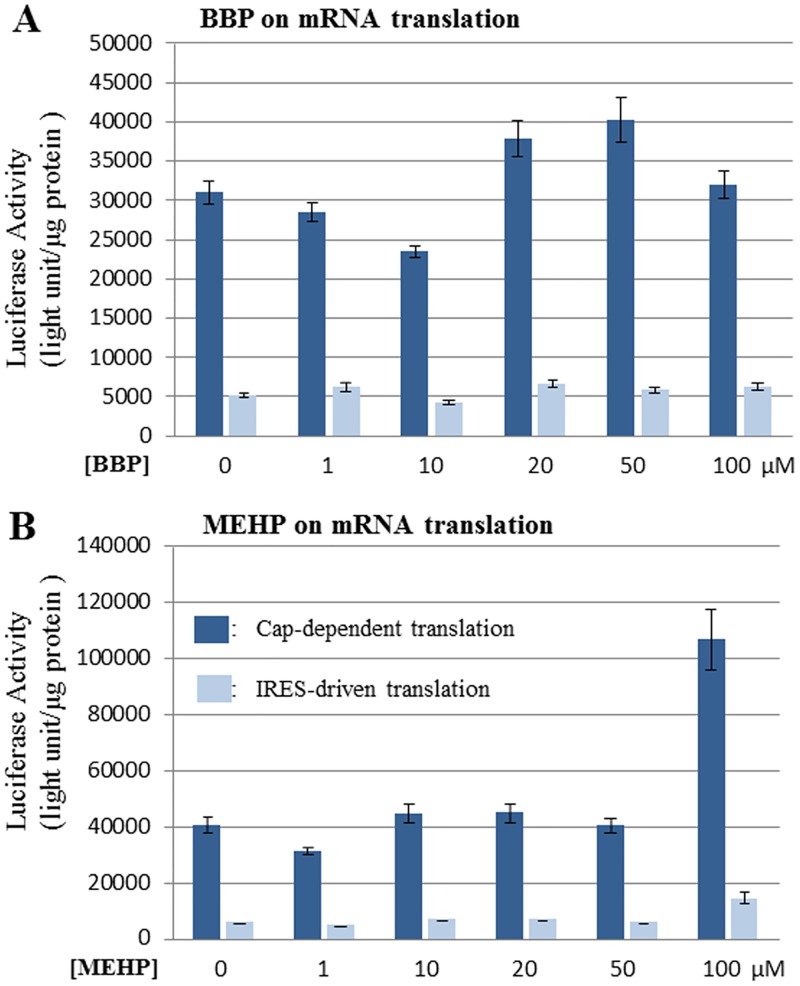
The effects of BBP and MEHP on mRNA translation in HT29 cells. HT29 cells were grown to 80% confluence and transfected with luciferase reporter. Eight hours later, cells were treated with BBP or MEHP for 24 hrs at the concentrations indicated. Equal volume of vehicle (methanol) was used as the control. Cells were harvested by washing once with cold PBS, and the cell lysate was prepared with PBL buffer (Promega) for the luciferase activity measurement using dual luciferase assay kit (Promega). **(A)** The effect of BBP on cap-dependent and IRES-driven translation; luciferase activity is the readout of translational activity. **(B)** The effect of MEHP on cap-dependent and IRES-driven translation. The experiments were repeated three times, and the means were shown with standard deviations.

To examine if phthalates affect mRNA translation in cancer cells, colon cancer cell HT-29 was exemplified in this study. The experiment was performed as same as those in Fig 2. It was found that BBP caused some perturbation on cap-dependent translation, but overall not showing much promoting or inhibitory effect; meanwhile BBP showed no effect on IRES-driven translation in HT29 cells (Fig 3A). On the other hand, MEHP exhibited no effect on both cap-dependent and IRES-driven translation at all concentrations up to 50μM. However, when MEHP concentration was increased to 100μM, both cap-dependent and IRES-driven translations were enhanced, among which cap-dependent translation was more sensitive (Fig 3B). The mechanism of such enhancing effect of MEHP in HT-29 cells is still under investigation. Nevertheless, the results from Fig 3 demonstrate the different effects of phthalates on mRNA translation in cancer cells, showing no effect or promoting mRNA translation.

### Modulation of eIFs is one of mechanisms underlying the effects of phthalate on mRNA translation

**Fig 4:**
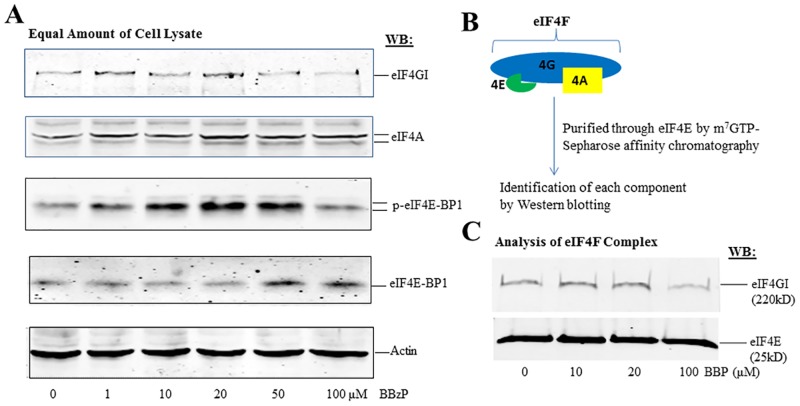
BBP modulates the expression and activity of eIFs in HEK-293T cells. HEK-293T cells were cultured to 80% confluency and treated with a series of concentrations of BBP for 24 hrs. The cells were harvested and washed once with cold PBS for the preparation of total cell lysate as described in the “materials and methods”. **(A)** Equal amounts of cell lysate (50μg) were analyzed by Western blot with antibodies against eIFs as indicated. **(B)** A diagram to show the purification of eIF4F complex by m^7^GTP-resin affinity chromatography. **(C)** Analysis of eIF4G and eIF4E in eIF4F complex by Western blot.

mRNA translation is regulated at numerous levels as described in the “Introduction”, here we focused on eIFs to explore the molecular mechanism underlying the effects of phthalates. After the cells treated with the same condition as that in Fig 2, total cell lysate was prepared for the analysis of eIFs by Western blotting (WB). eIFs involved in the binding of 5’-cap structure and the recruitment of mRNA into ribosome were specifically analyzed. As shown in Fig 4A, eIF-4A was not affected by BBP treatment; eIF4G was slightly elevated by increasing concentrations of BBP up to 20μM, followed by the decrease with 50μM and 100μM treatments, thus consistent with the changes in translation activity as shown in Fig 2B. Furthermore, the phosphorylation of eIF4E-BP was identified to be remarkably increased by the lower BBP concentrations up to 20μM, followed by the decrease with 50μM and 100μM treatments. Meanwhile, the non-phosphorylated eIF4E-BP was also found to be unaffected at lower concentrations up to 20μM but increased by higher concentrations of BBP at 50μM and 100μM. With the facts that phosphorylated eIF4E-BP promotes translation and non-phosphorylated eIF4E-BP inhibits translation, it can be considered that the expression and phosphorylation of eIF4E-BP is a key factor to mainly mediate the effect of BBP on translation.

eIF-4F is composed of eIF4E, eIF-4A, eIF4G, therefore becoming one of critical components to regulate mRNA translation rate. eIF4G shown in Fig 4A is the total eIF4G, it is not known how much is the bound form in eIF-4F. Thus, the active fraction of eIF4G in the eIF-4F complex was analyzed by a strategy as illustrated in Fig 4B. After the purification of eIF4F complex via the binding of eIF4E to m^7^GTP-resin, the components of eIF4F were analyzed by WB (Fig 4C). It was detected that the bound eIF-4G was slightly increased at 10μM and 20μM of BBP but decreased at 100μM of BBP under the constant amounts of eIF4E, therefore proving that the activity of eIF-4G is also an important factor to regulate the effect of BBP on mRNA translation.

**Fig 5:**
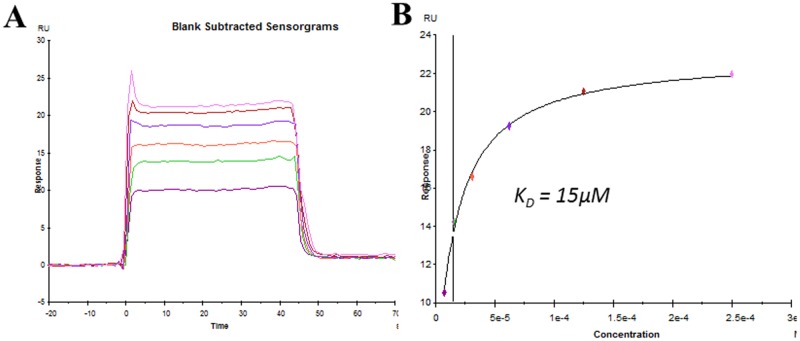
Measurement of binding of BBP to eIF4E by SPR. Purified eIF4E was immobilized to the Fc2 channel of a CM5 sensor chip as the ligand. The immobilization was performed through amine-coupling reaction. Fc1 channel was treated similarly but without protein as the control. Different concentrations of BBP were applied through Fc1 and Fc2 channels as the analyte to measure the binding. **(A)** Adjusted sensorgrams (Fc2-Fc1) showing the binding of eIF4E to various concentrations of BBP. **(B)** Calculation of binding affinity using steady-state fitting model.

It is also a critical question whether BBP directly targets eIFs or indirectly causes the modification of eIFs through other pathways, such as signal transduction. Like other small molecule antibiotics that target mRNA translation through direct binding to ribosome [43], the possibility of BBP binding to eIFs was examined by SPR method here. As shown in Fig 5, eIF4E was identified to show specific binding to BBP with the affinity at 15μM, which belongs to the medium strength of intramolecular interaction. This result suggested that the direct interaction of BBP with eIF-4E might play a broad role in mediating the effects of BBP on translation *in vivo*. In terms of the whole picture of translational factors targeted by phthalates, further experiments need to be done, for instance using phthalates as the ligands on SPR chips to screen for binding partners as the translational components. Alternatively mass spectrometry can be also utilized to identify phthalates in purified translational complexes.

### Normal and cancer cells respond to phthalate toxicity differently

**Fig 6:**
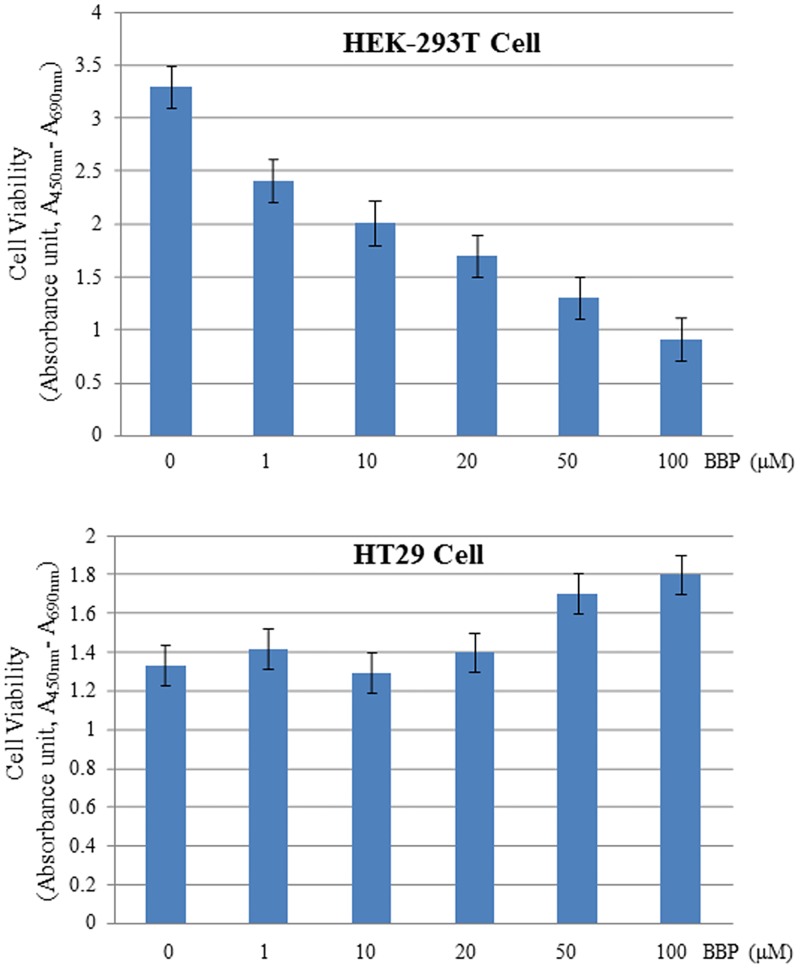
The effects of BBP on cell proliferation. Both HEK-293T and HT-29 cells were cultured to 80% confluency and treated with BBP at a series of concentrations as indicated. After the treatment for 24 hrs, the cell viability was measured as described in the “Materials and methods”. Each concentration point was repeated three times, and the means were shown with standard deviations.

Since mRNA translation is a fundamental process of gene expression to synthesize total cellular proteins, the effects of phthalates on mRNA translation should be reflected on some cellular processes. Here we specifically tested the cell viability to evaluate the toxicity of phthalates. As shown in Fig 6, BBP clearly inhibited the growth of normal HEK-293T cells in a concentration-dependent manner; whereas colon cancer cell HT-29 was not responsive or even slightly promoted by BBP, implying that phthalates might have general toxicity to normal cells but express growth-promoting or pro-survival effect on cancer cells. Such differential effects of phthalates on human normal and cancer cells may be partially due to the re-programming of mRNA translation machinery in cancer cells.

## Discussion

**Fig 7:**
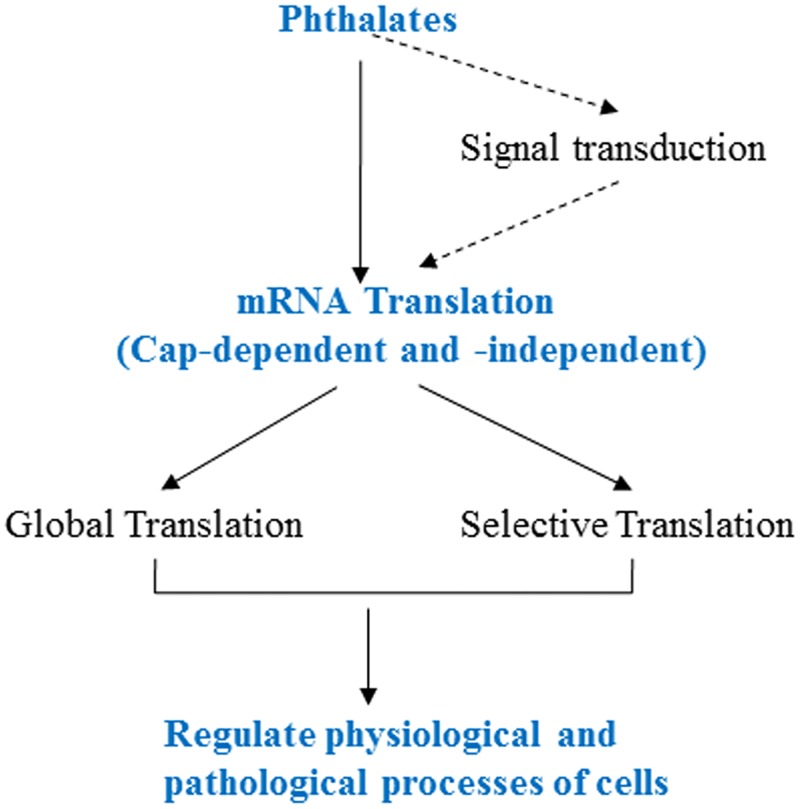
Working model of regulation of mRNA translation by phthalates. The dashed arrows indicate the pathway not studied in the paper.

This study reported for the first time that phthalates can directly inhibit mRNA translation *in vitro* and affect cap-dependent and -independent translation differentially *in vivo*. The modulation of eIF4E-BP and eIF-4G was identified to be a key molecular mechanism for the toxicity of phthalate. The overall working model is summarized in Fig 7, wherein the cap-dependent and -independent translations are differentially regulated by phthalates, leading to the different effects on global and selective mRNA translations. The synergistic effect between these two modes of translation will determine the cell fate in response to specific phthalate.

Furthermore, phthalate was found to inhibit normal cell growth but slightly promote cancer cell growth, suggesting that phthalates may express toxicity to damage normal tissues and to facilitate cancer cell progression, both of which are adverse effects to human health.

As an ongoing research, we will test more types of phthalate on their patterns to regulate mRNA translation. More phthalate sensitive cells (e.g., Leydig cell, Sertoli cell, Granulosa cell, small intestine epithelial cell, uterine epithelial cell, etc.) and cancer cell lines (e.g., liver, prostate, breast, cervical, and colon cancers) will also be examined. Hence, the specific effects of phthalates on mRNA translation in different cells can be identified. In addition, more translational factors and ribosomal components will be systematically screened, so that the overall picture of phthalate on mRNA translation can be elucidated. These investigations will further advance our understanding on the molecular mechanisms of phthalate-related disorders and diseases.

Since mRNA translational machinery is one of the most abundant molecular complexes in cells, identification of any of its components, such as eIFs, ribosomal proteins, and rRNAs, will facilitate the development of more sensitive biomarkers to evaluate phthalate toxicity, thus contributing to the prevention and diagnosis of phthalate-derived health problems.
